# Are SNP-Smoking Association Studies Needed in Controls? DNA Repair Gene Polymorphisms and Smoking Intensity

**DOI:** 10.1371/journal.pone.0129374

**Published:** 2015-05-27

**Authors:** Zoraida Verde, Luis Reinoso, Luis Miguel Chicharro, Pilar Resano, Ignacio Sánchez-Hernández, Jose Miguel Rodríguez González-Moro, Fernando Bandrés, Félix Gómez-Gallego, Catalina Santiago

**Affiliations:** 1 Department of Morphological Sciences and Biomedicine, Universidad Europea, Madrid, Spain; 2 Department of Occupational Health, Grupo Banco Popular, Madrid, Spain; 3 Department of Neumology, Hospital Guadalajara, Guadalajara, Spain; 4 Department of Neumology, Hospital Carlos III, Madrid, Spain; 5 Department of Neumology, Hospital Gregorio Marañón, Madrid, Spain; 6 Department of Toxicology and Health Sanitary, Universidad Complutense, Madrid, Spain; 7 School of Doctoral Studies & Research, Universidad Europea, Madrid, Spain; University of South Alabama Mitchell Cancer Institute, UNITED STATES

## Abstract

Variations in tobacco-related cancers, incidence and prevalence reflect differences in tobacco consumption in addition to genetic factors. Besides, genes related to lung cancer risk could be related to smoking behavior. Polymorphisms altering DNA repair capacity may lead to synergistic effects with tobacco carcinogen-induced lung cancer risk. Common problems in genetic association studies, such as presence of gene-by-environment (G x E) correlation in the population, may reduce the validity of these designs. The main purpose of this study was to evaluate the independence assumption for selected SNPs and smoking behaviour in a cohort of 320 healthy Spanish smokers. We found an association between the wild type alleles of *XRCC3* Thr241Met or KLC3 Lys751Gln and greater smoking intensity (OR = 12.98, 95% CI = 2.86–58.82 and OR=16.90, 95% CI=2.09-142.8; respectively). Although preliminary, the results of our study provide evidence that genetic variations in DNA-repair genes may influence both smoking habits and the development of lung cancer. Population-specific G x E studies should be carried out when genetic and environmental factors interact to cause the disease.

## Introduction

Smoking is the single biggest preventable cause of death in contemporary societies [1]. Its consumption results in greater incidence of cardiovascular disease, pulmonary disease and many cancers [2].

Cigarette smoke contains large quantities of carcinogens, including polycyclic aromatic hydrocarbons, which damage DNA by covalent binding or oxidation [3]. Although cigarette smoking is the major cause of lung cancer, only a small fraction of smokers develop smoking-related lung cancer, suggesting that other causes, including genetic susceptibility, may contribute to the variation in individual lung cancer risk [4–6]. This genetic susceptibility may be due, in part, to genetically determined variation in carcinogen metabolism [7] and/or in the capacity of DNA repair [8–10]. DNA-repair activities are essential for the protection of the genome from environmental damage such as tobacco smoke [11]. However, contradictory results are often reported by various studies, making it difficult to interpret them [12,13]. Approximately 160 genes mediate DNA repair have been found in human cells [14]. Several polymorphisms in DNA repair genes contribute to genetic instability and error accumulation due to reduced protein activity being associated to relatively risk of lung cancer in Caucasian population [15,16,17,18]. The NER (nucleotide excision repair) pathway repairs DNA damage caused by the tobacco-related carcinogen benzo(a)pyrene, while the BER (base excision repair) pathway repairs DNA caused by reactive oxygen species (ROS) results from cigarette smoke [19]. In addition, DSBR (double strand break repair) pathway is the responsible for repairing double-strand breaks produced by exogenous agents such as environmental carcinogens present in tobacco smoke and endogenous generated ROS [20].

Variants in the genes encoding aforementioned proteins are very common in the population. Most of studies have analyzed genetic polymorphisms in *XPD*, *XRCC1*, *APEX1* and *XRCC3* genes. The presence of the alleles 312Asn and 751Gln of *XPD* has been associated with risk of lung cancer in Caucasian individuals [15,16]. BER genes repair DNA damage from oxidation, deamination and ring fragmentation [21]. *XRCC1* Arg399Gln polymorphism and lung cancer risk has been analyzed in relatively high number of studies [22,23,24,18]. XRCC3 participates in repair DNA-double strand break via homologous recombination, the polymorphism of *XRCC3* Thr241Met has been indicated to be involved in the development of some cancers [25]. In addition, APE1 protein plays a role in repairing abasic sites [26]. Single-nucleotide polymorphisms of the *APE1* gene have been demonstrated to be involved in carcinogenesis. However, the association between *APE1* Asp148Glu polymorphism and lung cancer risk remains inconclusive in Caucasian population [27].

Variants in DNA repair genes modulate DNA repair activity in smokers and therefore could alter cancer risk [28]. Inconsistent results have been published possibly due to low statistical power, false-positive results, heterogeneity across studies populations, failure to consider environmental exposures or publication bias [29].

Variations in tobacco-related cancers, incidence and prevalence reflect differences in tobacco consumption in addition to genetic factors. Besides, genes related to lung cancer risk could be related to smoking behavior.

Polymorphisms altering DNA repair capacity may lead to synergistic effects with tobacco carcinogen-induced lung cancer risk [30].

Published control group data on the associations of interest for gene-by-environment (G×E) interaction are limited [31]. Common problems in genetic association studies, such as presence of G x E correlation in the population, may reduce the validity of these designs. The main purpose of this study was to evaluate the independent assumption for selected SNPs and smoking behaviour in a cohort of healthy Spanish smokers.

Polymorphisms of interest were single nucleotide changes (SNPs) in *XRCC1* (Arg399Gln) [rs25487], *APEX1* (Asp148Glu) [rs1130409], *XRCC3* (Thr241Met) [rs861539], *XPD* (Asp312Asn) [rs1799793] and (Lys751Gln) [rs13181]. Lung cancer susceptibility has been examined in numerous epidemiological studies that have investigated the association between the development of the pathology and variants in candidate genes. We have analysed the aforementioned polymorphisms attending to previous publications and prevalence in Caucasian population [23,24,32,33,34]. We have selected five functional polymorphisms that have been considered as lung cancer risk factors in Caucasian population in order to replicate in a healthy smokers population.

## Methods

### Ethics statement

Approval was obtained from the local Ethics Committee (Hospital Carlos III, Madrid) and all patients provided written informed consent. The study was in accordance with the Helsinki Declaration.

### Subjects

Three hundred and twenty healthy smokers (all of Caucasian (Spanish) descent for ≥3 generations) between 25 and 65 years of age were recruited from the Health and Safety Committee of Banco Popular, Madrid (Spain); Department of Neumology, Hospital Carlos III, Madrid, Spain; and Department of Neumology, Hospital Gregorio Marañón, Madrid, Spain; from 2010 to 2013. Eligible participants were 25–65 years old and reported smoking ≥ 1 cigarette per day for ≥ 5 years. Exclusion criteria included suffer from any illness related to smoking.

### Phenotype assessment

All participants completed a questionnaire regarding demographic characteristics, smoking habits, self-reported cigarettes per day (CPD), the number of years the person had smoked and pack years smoked (PYS). The PYS is used to describe the number of cigarettes a person has smoked over a lifetime and it is calculated by multiplying the number of cigarettes smoked per day by the number of years the person has smoked and divided by 20. Nicotine dependence was assessed with the Fagerstrom Test for Nicotine Dependence (FTND) [35]. In addition, CO levels and lung function (spirometry) were measured in each participant. We divided the smokers attending CO levels (ppm) in: very light smoker (0–6), light smoker (7–10), smoker (11–20) and heavy smoker (>20). In order to check CPD reported we measure cotinine levels in 30% of participants.

### Genotype assessment

Peripheral blood samples were obtained by venipuncture. Blood leukocyte DNA was extracted using a standard phenol chloroform protocol. The DNA isolation and genotype analyses were performed in the Biomedicine laboratory at the Universidad Europea, Madrid (Spain). The study followed recommendations for replicating genotype-phenotype association studies [36]: genotyping was performed specifically for research purposes, and the researchers in charge of genotyping were totally blinded to the participants’ identities (blood and DNA samples were tracked solely with bar-coding and personal identities were only made available to the main study researcher who was not involved in actual genotyping). The DNA samples were diluted with sterile water and stored at -20°C until analysis.

Genotyping was performed by Real-time PCR and Taqman probes with a Step One Real-Time PCR System (Applied Biosystems, Foster City, CA).

### Statistical analysis

We compared smoking phenotypes among the different genotypes and combination of genotypes with the unpaired Student’s *t*-test. We used the χ^2^ test to assess deviations of genotype distributions from the Hardy-Weinberg equilibrium (HWE). Logistic regression analysis was carried out to calculate G-E interactions between smoking habits and genotypes or genotype combinations adjusted for different covariates (i.e., age and gender). All statistical analyses were adjusted for multiple comparisons using the Bonferroni method, in which the threshold *P*-value is obtained by dividing 0.05 by the number of tests. All analyses were performed with the PASW/SPSS Statistics 20.0 (SPSS Inc, Chicago, IL) program.

## Results

The study included 320 healthy current smokers, 55.00% men, all Caucasian with a mean age of 48.64 years (SD = 13.48). On average, they had been smoking for 24.57 years (SD = 10.88). The CPD and PYS ranged from 5 to 70 and 2 to 175 with an overall mean of 17.60 (10.59) cigarettes/day and 28.16 (24.44) PYS, respectively.

**Fig 1:**
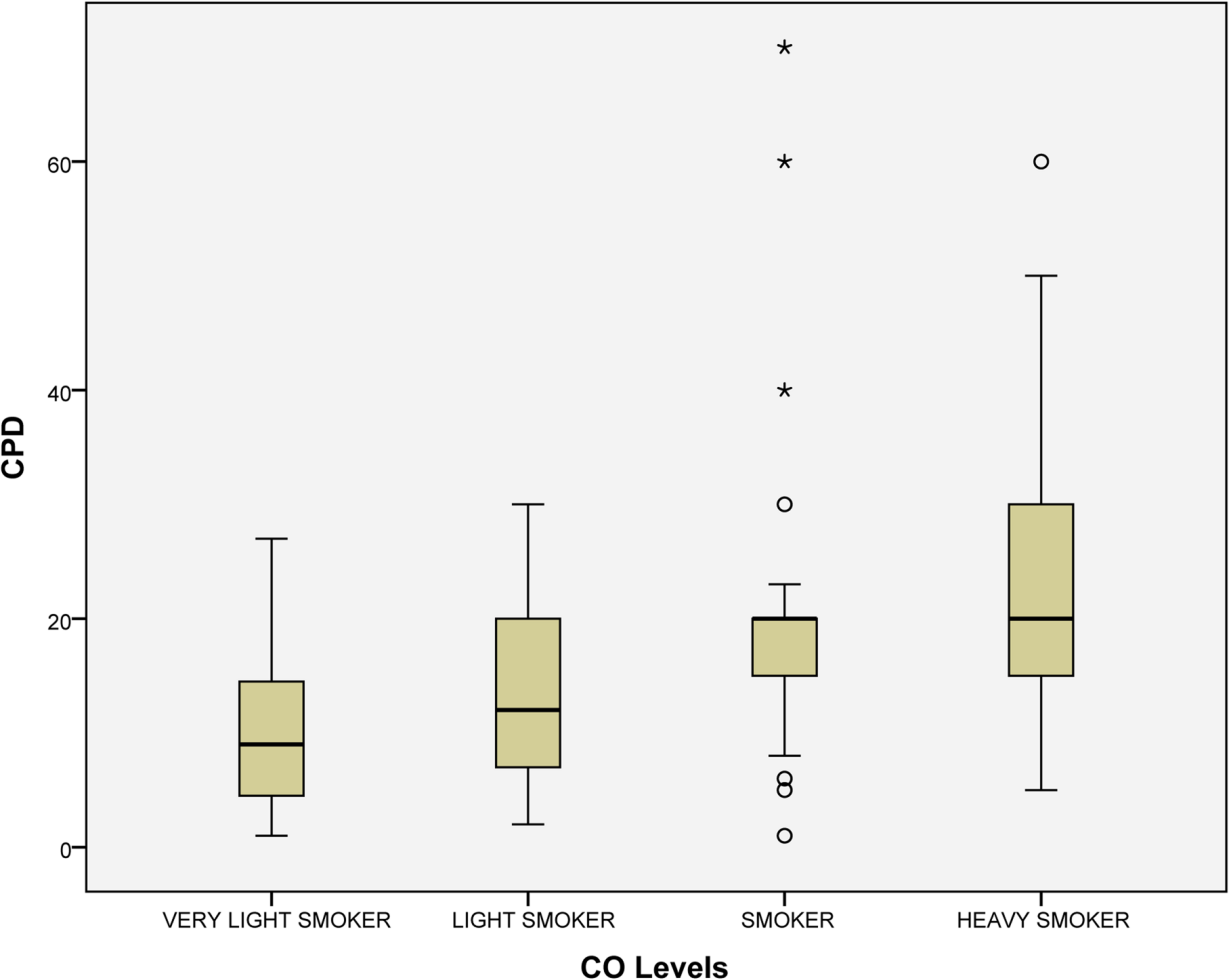
Association of CO levels and cigarettes reported. Note: The smokers were divided considering CO levels (ppm) into: very light smoker (0–6), light smoker (7–10), smoker (11–20) and heavy smoker (>20). Abbreviations: CPD, cigarettes per day.

**Fig 2:**
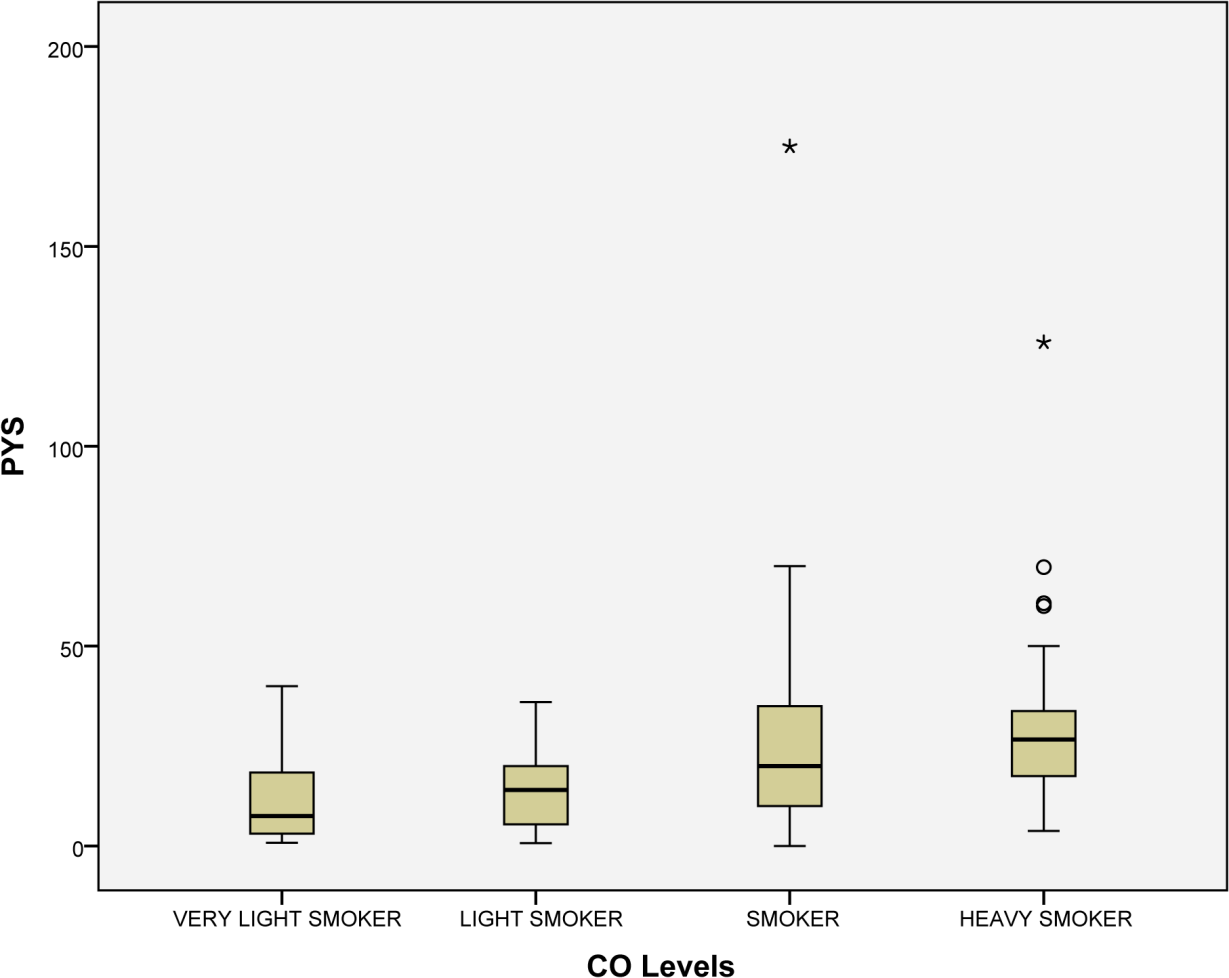
Association of CO levels and packs year smoked. Note: The smokers were divided considering CO levels (ppm) into: very light smoker (0–6), light smoker (7–10), smoker (11–20) and heavy smoker (>20). Abbreviations: PYS, packs year smoked.

In order to check the number of CPD reported, the levels of CO (ppm) expired were tested in each smoker, resulting the following percentage in each category: very light smoker (19.6%), light smoker (15.6%), smoker (39.2%), and heavy smoker (25.6%). Statistically significant differences were found (P<0.001) among the following categories: very light smoker 9.77 CPD (SD = 6.52), light smoker 12.86 CPD (SD = 7.11), smoker 19.58 CPD (SD = 10.43), and heavy smoker 22.94 CPD (SD = 10.69). In addition, significant differences were found between PYS and CO levels (*P*<0.001): very light smoker 11.52 PYS (SD = 10.88), light smoker 14.91 PYS (SD = 10.38), smoker 24.38 PYS (SD = 22.06), and heavy smoker 29.43 PYS (SD = 19.68) (Figs 1 and 2).

All genetic polymorphisms studied in the population were in Hardy Weinberg Equilibrium (HWE) except *XRCC1* Arg399Gln polymorphism (*P* = 0.02). No differences in genotype distribution between females and males were observed (data not shown). Genotype frequencies were, respectively: *XRCC1* Arg399Arg = 42.6%, Arg399Glu = 52.7, Glu399Glu = 4.7%; *APEX1* Asp148Asp = 31.1, Asp148Gln = 45.1, Gln148Gln = 23.9; *XRCC3* Thr241Thr = 35.3, Thr241Met = 48.0, Met241Met = 16.7; *ERCC2* Asp312Asp = 43.9, Asp312Asn = 45.2, Asn312Asn = 10.9; *KLC3* Lys751Lys = 41.8, Lys751Gln = 45.1 and Gln751Gln = 13.1; showed similar frequency as reported in previous studies in Spanish population [17].

Analyses focused on associations with genotype categorized using a recessive model (i.e. homozygotes of the most common allele plus heterozygotes were the referent group, compared to homozygotes of the minor allele).

### Association between DNA repair variants and smoking behaviour

Genotype-smoking associations between *XRCC3* Thr241Met and smoking intensity (PYS) and years smoking were found (*P* = 0.001 and *P* = 0.004, respectively). Logistic regression analysis showed an association between the most common allele of *XRCC3 Thr241Met* and greater smoking intensity (OR = 12.98, 95% CI = 2.86–58.82) and more years smoking (OR = 20.66, 95% CI = 2.57–166.62).

In addition, we found an association between the most common allele of *KLC3* Lys751Gln and greater smoking intensity (OR = 16.90, 95% CI = 2.09–142.8) and years smoking (OR = 12.19, 95% CI = 1.49–100.00). For the remaining genotypes we didn´t find a statistically association with smoking habits. For additional data see Table 1.

**Table 1:** Relationship between genotypes and smoking behaviour.

	N	FTND		CPD		PYS		YS	
		Mean (SD)	^*a*^ *P-*value	Mean (SD)	^*a*^ *P-*value	Mean (SD)	^*a*^ *P-*value	Mean (SD)	^*a*^ *P-*value
***XRCC1* Arg399Gln**									
Arg/Arg,Arg/Gln	297	4.2 (2.8)	0.952	17.6 (10.7)	0.895	27.4 (24.1)	0.562	24.4 (10.8)	0.998
Gln/Gln ^b^	23	5.2 (2.8)		16.2 (8.4)		36.8 (28.3)		31.0 (11.5)	
***APEX1* Asp148Glu**									
Asp/Asp,Asp/Glu	243	4.4 (2.8)	0.804	16.2 (10.8)	0.608	29.5 (25.7)	0.083	24.6 (10.9)	0.267
Glu/Glu ^b^	77	3.9 (2.9)		16.0 (9.8)		24.8 (20.9)		24.2 (11.0)	
***XRCC3* Thr241Met**									
Thr/Thr, Thr/Mer	266	4.5 (2.8)	0.076	18.2 (10.4)	0.629	29.2 (24.2)	0.001	25.1 (10.9)	0.004
Met/Met ^b^	54	3.2 (2.6)		15.1 (11.0)		23.5 (25.5)		22.1 (10.4)	
***ERCC2* Asp312Asn**									
Asp/Asp,Asp/Asn	284	4.2 (2.8)	0.167	17.9 (10.6)	0.322	28.7 (24.7)	0.110	26.7 (10.8)	0.242
Asn/Asn ^b^	36	4.1 (2.5)		15.6 (11.1)		24.3 (24.1)		22.7 (11.5)	
***KLC3* Lys751Gln**									
Lys/Lys, Lys/Gln	278	4.3 (2.9)	0.166	17.9 (10.5)	0.342	28.4 (24.1)	0.008	25.1 (10.7)	0.02
Gln/Gln ^b^	42	3.8 (2.1)		15.5 (11.1)		27.4 (27.1)		21.9 (11.9)	
**Number of risk alleles**									
0–2	85	3.5 (2.6)	0.065	15.7 (12.0)	0.714	27.7 (30.1)	<0.001	22.9 (11.4)	<0.001
3–4	235	4.6 (2.8)		18.0 (9.4)		28.7 (21.0)		25.2 (10.6)	

FTND, Fagerstrom test for nicotine dependence; CPD, cigarettes per day; PYS, pack years smoked; YS, years smoking.

^a^ Adjusted *P* value by age and gender.

^b^ Reference allele.

Under the assumption that the combination of polymorphism can have additive or more than additive effects, the combination of two significant variants was investigated.

We analysed the combination of the most common alleles of *KLC3* Lys751Gln and *XRCC3* Thr241Met. When the study population was categorized according to the number of risk alleles, smoking habits (PYS) and years smoking were statistically significantly increased in individuals bearing three-four risk alleles (*P*<0.001) in both.

## Discussion

The case-only study design has been increasingly used to estimate the magnitude of statistical interaction between 2 measured exposures with respect to a given outcome, most commonly a genetic and an environmental exposure [37].

However, results from the case-only design can be misleading due to, at least, two problems. First, the assumption of independence of genetic and environmental factors, meaning that when genetic and environmental factors are associated, the design may wrongly lead to the conclusion that interaction exists [38]. Second, a statistical interaction does not guarantee a biological relationship when genetic and environmental factors interact to cause the disease. The independent effect of either exposure, or interaction on the additive scale, cannot be estimated.

Little empirical work has been conducted to quantitatively assess the magnitude of control-only associations between DNA repair gene variations and smoking. Moreover, to our knowledge, only a few studies have investigated the associations between in vitro-induced DNA adduct levels and genetic variations in DNA repair genes in normal cells from healthy individuals.

Although asking for the number of CPD is currently accepted as the gold standard measure of exposure, it may not be a good indicator. There are many factors that alter the real exposure, such as individual variability, gender, type of cigarette or the lack of precision reporting the number of CPD. There is wide recognition that a proportion of current smokers underestimates tobacco consumption or even denies smoking entirely. In our sample, CPD reported by smokers were in accordance to CO levels tested, so the population was correctly phenotyped.

Expired CO levels correlate closely with specific cotinine assays and reliably reflect smoking habits [39]. Our results showed that there was a significantly positive association between daily consumption of cigarettes and CO levels, and between PYS and CO levels in healthy smokers. We considered strict criteria for phenotypic measures. Despite of smoking may seem to be a simple phenotype with measurable parameters as cigarette smoked per day, describing a reliable phenotype could be a difficult problem in scientific research because subjective estimations are used instead of real measures [40].

Analysing the effect of DNA repair variants in smoking behaviour we found associations between *XRCC3* Thr241Met and *KLC3* Lys751Gln variants and smoking habits in Spanish population. Furthermore, when we investigated the combination of *KLC3* Lys751 and *XRCC3* Thr241 alleles, a highly significant association with smoking was observed in the subjects carrying three or more risk alleles. PYS showed the highest association, thus PYS is a feasible way to measure the amount a person has smoked over a long period of time [41].

Smoking amount (PYS) may be causally associated with the most common alleles of *XRCC3* Thr241Met and *KLC3* Lys751Gln. In addition, smokers with the *XRCC3* Thr241or *KLC3* Lys751 alleles presented more nicotine addiction measured by FTND and more years smoking. There is evidence that *XRCC1* Arg399Gln, *KLC3* Lys751Gln and *XRCC3* Thr241Met variants are functional [42,43]. Several authors have analysed the effect of different combinations of DNA repair SNPs and the levels of DNA adducts [43–47]. Inverse significant associations on DNA adducts have been detected in *XRCC3* Met241Met carriers [46]. In the same way, other authors have also been described for *XRCC3* Met241 carriers an association with reduced repair of X-ray-induced cytogenetic damage measured by chromatid aberrations [43,47]. The *XRCC3* Thr241Met polymorphism is a non-conservative substitution with possible biological implications for the function of the enzyme and/or the interaction with others DNA repairing proteins. Amino acid variants in different domains of DNA repair proteins may not only affect different protein interactions, resulting in the expression of different phenotypes [48], but also the same polymorphism may have divergent effects on different DNA repair pathways and on different types of DNA damage [43].

Attending our results, across SNPs, *XRCC3* Thr241Met and *KLC3* Lys751Gln polymorphisms could be related to nicotine addiction measured as smoking amount (PYS) or years smoking.

In a meta-analysis Hodgson et al. reported similar associations as those we found [31]. There is some evidence that variation in DNA repair activity may affect neurological and/or respiratory outcomes, which could in turn affect smoking behaviour [49,50]. Different aspects of smoking behaviour (smoking initiation, smoking cessation, intensity etc.) operate through multiple overlapping pathways, therefore would not be expected to be identically affected by DNA repair variation [43].

Population stratification could have contributed to the heterogeneity in G-E associations. Variant alleles are found at different frequencies in different ethnic groups within the same study, and smoking behaviour may also differ by ethnicity [31]. Moreover most of the studies of G-E interaction with smoking amount information are lung cancer studies [51]. G-E associations in controls may be population-specific.

*Hung and cols*., published typical problems regarding investigations of G-E interactions, in particular, the fact that among the generally negative results, some seemingly noteworthy associations are identified in subgroups of subjects who are defined on the basis of their tumor histology or smoking habits. In some cases exists the probability that the associations found are attributable to chance (i.e., false positives). The challenge is to distinguish the false-positive associations from the true positives [52]. Several authors propose a simple Bayesian approach that is based on the estimation of a prior probability and the calculation of posterior probability [52]. Meta-analysis or functional analysis can be extremely useful for obtaining prior estimates [51,53].

A weakness of our study was the low sample size of healthy smokers, yet we believe this can be partly overcome by the fact that our population is homogeneous, not stratified and well defined in terms of phenotype assessment.

Although preliminary, the results of our study provide evidence that several genetic variations in DNA-repair genes may influence not only smoking habits but also the development of lung cancer.

To our knowledge, there are no previous studies G-E on DNA repair genes polymorphisms and smoking habits in healthy Spanish population. The results of our study are overall consistent, as they comply with the following published guidelines [54]: the smoking phenotypes and the study outcome were properly measured and accurately recorded by a researcher who was blind to the genetic information and was an expert in the area (Neumologist), we corrected all statistical inferences for multiple comparisons (Bonferroni’s criteria for the p-values); and the results are overall in accordance with previous research in the field. Studies with more sophisticated designs (including more appropriate smoking phenotype measurements and representative population) are required even with the risk of smaller sample size [21, 55].
